# A novel approach to minimal reservoir computing

**DOI:** 10.1038/s41598-023-39886-w

**Published:** 2023-08-10

**Authors:** Haochun Ma, Davide Prosperino, Christoph Räth

**Affiliations:** 1 Department of Physics, Ludwig-Maximilians-Universität, Schellingstraße 4, 80799 Munich, Germany; 2 Deutsches Zentrum für Luft- und Raumfahrt (DLR), Institut für KI Sicherheit, Wilhelm-Runge-Straße 10, 89081 Ulm, Germany

**Keywords:** Complex networks, Information theory and computation

## Abstract

Reservoir computers are powerful machine learning algorithms for predicting nonlinear systems. Unlike traditional feedforward neural networks, they work on small training data sets, operate with linear optimization, and therefore require minimal computational resources. However, the traditional reservoir computer uses random matrices to define the underlying recurrent neural network and has a large number of hyperparameters that need to be optimized. Recent approaches show that randomness can be taken out by running regressions on a large library of linear and nonlinear combinations constructed from the input data and their time lags and polynomials thereof. However, for high-dimensional and nonlinear data, the number of these combinations explodes. Here, we show that a few simple changes to the traditional reservoir computer architecture further minimizing computational resources lead to significant and robust improvements in short- and long-term predictive performances compared to similar models while requiring minimal sizes of training data sets.

## Introduction

The prediction of complex dynamic systems is a key challenge across various disciplines in science, engineering, and economics^1^. While machine learning approaches, like generative adversarial networks, can provide sensible predictions^2^, difficulties with vast data requirements, the large number of hyperparameters, and lack of interpretability limit their usefulness in some scientific applications^3^. However, it is required to fundamentally understand how, when, and why the models are working to prevent the risk of misinterpreting the results if deeper methodological knowledge is missing^4^.

In the context of complex systems research, reservoir computers (RCs)^5,6^ have emerged for predicting the dynamics of chaotic systems. The core of the model is a fixed reservoir, which is usually constructed randomly^7–9^. The input data is fed into the nodes of the reservoir and solely the weights of the readout layer, which transform the reservoir response to output variables, are subject to optimization via linear regression. This makes the learning extremely fast and comparatively transparent. However, this approach can be hit-or-miss, and it is hardly possible to know a priori how the topology of the reservoir will affect the performance^10–12^.

Recent research has emerged on algorithms which do not require randomness. They are built around regressions^13^ on large libraries of linear and nonlinear combinations constructed from the data observations and their time lags, such as next generation reservoir computers (NG-RCs)^14^ or sparse identification of nonlinear dynamics (SINDy)^15^. These algorithms are built around nonlinear vector autogression (NVAR)^16^ and the mathematical fact that a powerful universal approximator can be constructed by using an RC with a linear activation function^17,18^.

The model we present in this paper is based on the same mathematical principles — but instead of getting rid of the traditional reservoir architecture altogether, we take an intermediate step and make only a few simple changes: we restructure the input weights so that all coordinate combinations are fed separately into the reservoir. Additionally, we remove the randomness of the reservoir by replacing it with a block-diagonal matrix of blocks of ones. Instead of introducing the nonlinearity in the activation function, we add higher orders of the reservoir states in the readout.

Using the example of synthetic, chaotic systems, and in particular the Lorenz system, we show that these alterations lead to excellent short- and long-term predictions that significantly outperform traditional RC, NG-RC, and SINDy. While prediction performance is often evaluated visually, we use three quantitative measures: the largest Lyapunov exponent, the correlation dimension, and the forecast horizon. We also validate the robustness of our results by using multiple attractor starting points, different training data sizes and discretizations.

## Results

**Figure 1:**
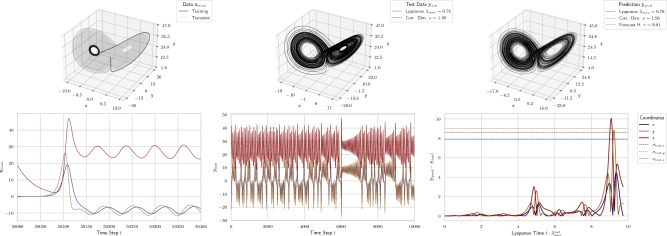
Prediction on a minimal training data set of the Lorenz system. The first column shows the attractor (top) and the trajectories (bottom) of the 400 training data points (and the discarded transient). The second column shows the attractor and the trajectories of the test data. The third column shows the attractor (top) and the absolute prediction error (bottom) of the prediction. The dashed lines indicate the standard deviations of the three components of the test data.

In this work, we show how small changes to the traditional RC architecture can significantly improve its prediction capability of chaotic systems especially for low data requirements. Therefore, similar to Gauthier et al.,^14^ we use the minimal data setup for the Lorenz system with a discretization of $dt{=}0.025$ and $T_{train}{=}400$ training data points.

The minimal possible architecture would be a spectral radius $\rho ^*{=}0$ and block-size $b{=}1$, for which our RC reduces to the case described by Gonon and Ortega.^17^ Here, we do not have a reservoir and directly feed the input data to the readout and perform a Ridge regression. While we find this parametrization to be capable of reasonable predictions, a few minor alterations increase the performance significantly.

The standard RC architecture used in this work has block-size $b{=}3$, spectral radius $\rho ^*{=}0.1$, and a nonlinearity degree $\eta {=}2$. This equals 36 variables per coordinate. The results of this setup are illustrated in Fig. 1.

**Figure 2:**
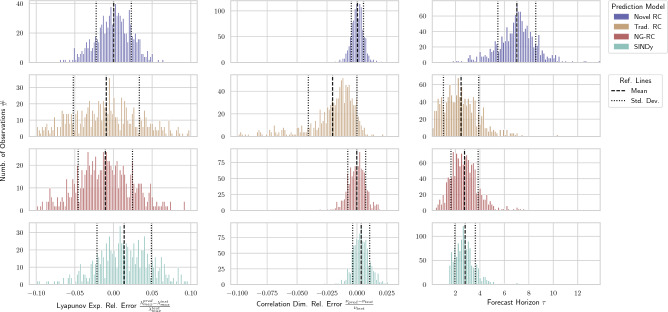
Prediction measures (columns) of different models (rows) for 1000 different starting points on the Lorenz attractor ($dt{=}0.025$, $T_{train}{=}400$). For the correlation dimension and the Lyapunov exponent we calculate the relative error to the respective test data. The mean and standard deviation of each distribution is denoted by a dashed and dotted black line, respectively.

In order to obtain robust results we repeat the analysis for $1000$ different starting points on the attractor and compare the prediction performance to the other models. In Fig. 2 we see that the novel RC architecture significantly outperforms them with regards to short-term predictions with an average forecast horizon of ${\sim }7.0$ Lyapunov times — this is ${\sim }2.5$ times more than the averages of the other models. The long-term prediction is also slightly better as the average relative errors of the correlation dimension and the Lyapunov exponent are ${\sim }3.5 \cdot 10^{-4}$, respectively — this is ${\sim }9.0$ and ${\sim }39.7$ times smaller than the averages of other models. The traditional RC has generally more widely distributed errors due to its randomness.

**Figure 3:**
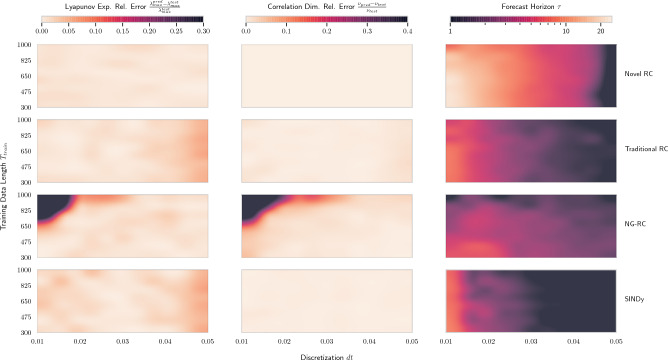
Prediction measures (columns) of different models (rows) for different discretizations and lengths of training data of the Lorenz system. We vary the discretization (x-axis) and the length (y-axis) of the training data between (0.01, 0.05) and (300, 1000), respectively. For the correlation dimension and the Lyapunov exponent we calculate the relative error to the respective test data. Note that the forecast horizon has a logarithmic color scale. Each value in the heatmaps is the average over 100 variations of attractor starting points.

We verify the robustness of our novel RC to variations in discretization and length of training data. In Fig. 3 we observe that it is quite robust and as expected, performs significantly better than comparable models especially with regards to short-term prediction. Here, we only see a decline in prediction performance for coarse discretizations $dt{>}0.045$. The robustness of the long-term prediction is similar to traditional RC and SINDy. Interestingly, we see a decline in performance of NG-RC for larger training lengths $T_{train}{>}700$ and finer discretizations $dt{<}0.02$. Furthermore, we find out model to be reasonably robust to changes in hyperparameters and noise up to a signal-to-noise ratio of ${\sim }38\text {dB}$.

Furthermore, we analyze the prediction performance of our model on different chaotic systems, which have different nonlinear behavior. We choose the models so that we can understand the inner workings of our RC better. For example, the Halvorsen system has only quadratic nonlinearities with no interacting coordinates and hence the input matrix only needs the first three blocks (which represent the distinct coordinates). Another example to point out is the Rabinovich-Fabrikant system, which has cubic nonlinearities. Here, we see that a nonlinearity degree of $\eta {\ge }3$ is necessary for a reasonable prediction. The model parameters and the prediction measures for the different systems are illustrated in Table 1.

## Discussion

In this work, we present a novel RC architecture that outperforms comparable methods in terms of short- and long-term predictions while requiring similarly minimal training datasets and computational power. The architecture is modified by restructuring the input weights and reservoir such that combinations of input data coordinates are fed separately into the reservoir. Therefore, we use a block-diagonal matrix of ones as the reservoir, which acts as an averaging operator for the reservoir states at each update step. Similar to average pooling layers in other machine learning methods, this can be interpreted as a way to primarily “extract” features that are more robust^19^. It also takes out the randomness of traditional RC. Instead of using a nonlinear activation function to create the reservoir states, we capture the nonlinearity of the data in the readout layer by appending higher orders of the reservoir states before the Ridge regression. We find that these changes lead to a significant improvement in the short- and long-term predictions of chaotic systems in comparison to models such as the traditional RC, NG-RC, and SINDy. In order to evaluate the prediction performance, we compute the largest Lyapunov exponent, the correlation dimension, and the prediction horizon.

This work can be extended in many directions. For example, the generation of the reservoir states can be explored to understand what the RC actually learns. In our modified architecture, the states are constructed by mixing the average of the past data with the new data with different “proportions”. Therefore, methods for constructing the reservoir states, such as the exponentially weighted moving average (EWMA) of the data^20^, should be explored. Related to this, the design of the readout is also an interesting topic to look into. Similarly to NG-RC and SINDy, nonlinear functions could be applied and appended to the reservoir states in order to capture more complex structures in the data.

Another study can be conducted on how the elimination of randomness from RC-like models affects their capabilities, e.g., information processing capacity^21^ or multifunctionality^22^.

Furthermore, the applicability to high-dimensional and highly nonlinear data can be analyzed and compared with models relying on large feature libraries, such as NG-RC and SINDy. Since the number of variables scales less rapidly in our architecture, it would be relevant to see how much computational power can be saved, especially for hardware RCs.

Moreover, the model can be tested on real-world examples from different disciplines to produce reliable short- and long-term predictions, especially in cases where training data is scarce and expensive.

## Methods

### Reservoir computers

A reservoir computer (RC)^5,23,24^ is an artificial recurrent neural network (RNN) that relies on a static network called *reservoir*. The term static means that, unlike other RNN approaches, the reservoir remains fixed once the network is constructed. The same is true for the input weights. Therefore, the RC is computationally very efficient since the training process only involves optimizing the output layer. As a result, fast training and high model dimensionality are computationally feasible, making RC well suited for complex real-world applications.

In the following we describe the individual components of the architecture and the modifications that we propose. To make the following section more understandable we introduce them in a high-level summary:

1. *Input weights*: the input weights ${\textbf{W}}_{in}$ are designed so that each combination of the coordinates of the data is fed into the reservoir separately.
2. *Reservoir*: the reservoir ${\textbf{A}}$ is chosen as a block-diagonal matrix consisting of matrices of ones with size *b*.
3. *Reservoir states*: we do not use a nonlinear activation function in order to construct the reservoir states ${\textbf{r}}(t)$. Hence the iterative update equation reduces to:
  1 $$\begin{aligned} {\textbf{r}}(t+1) = {\textbf{A}} \cdot {\textbf{r}}(t) + {\textbf{W}}_{in} \cdot {\textbf{u}}(t) \,, \end{aligned}$$
  where ${\textbf{u}}(t)$ denotes the training data at time *t*.
4. *Readout*: instead of only inserting the squared reservoir states^25^, our generalized states $\tilde{{\textbf{r}}}$ contain all orders up to a nonlinearity degree $\eta$:
  2 $$\begin{aligned} \tilde{{\textbf{r}}} = \left\{ {\textbf{r}}, {\textbf{r}}^2, \dots , {\textbf{r}}^{\eta - 1}, {\textbf{r}}^\eta \right\} \,. \end{aligned}$$
5. *Training and Prediction*: as in traditional RCs, we stack the training data ${\textbf{u}}$ and the corresponding reservoir states $\tilde{{\textbf{r}}}$ to matrices ${\textbf{U}}$ and $\tilde{{\textbf{R}}}$ respectively. We then solve the equation ${\textbf{W}}_{out}\cdot \tilde{{\textbf{R}}} = {\textbf{U}}$ by using Ridge regression^26^ resulting in:
  3 $$\begin{aligned} {\textbf {W}}_{out} = {\textbf{U}}\cdot \tilde{{\textbf{R}}}^T \left( \tilde{{\textbf{R}}} \cdot \tilde{{\textbf{R}}}^T + \beta \,{\textbf{I}}\right) ^{-1} \,, \end{aligned}$$
  where $\beta {=}10^{-5}$ is the regularization constant that prevents overfitting and ${\textbf{I}}$ denotes the identity matrix. The prediction procedure of the reservoir states also stays the same (with the adjusted update equation):
  4 $$\begin{aligned} {\textbf{r}}(t+1) = {\textbf{A}} \cdot {\textbf{r}}(t) + {\textbf{W}}_{in} \cdot {\textbf{W}}_{out} \cdot \tilde{{\textbf{r}}}(t) \,. \end{aligned}$$
  Note that the reservoir ${\textbf{A}}$ only acts on the “simple” reservoir state ${\textbf{r}}$, while the second summand acting on ${\textbf{W}}_{out}$ is $\tilde{{\textbf{r}}}$ containing all the nonlinear powers. The predicted time series ${\textbf{y}}(t)$ can then be obtained by the multiplication:
  5 $$\begin{aligned} {\textbf{y}}(t) = {\textbf {W}}_{out} \cdot \tilde{{\textbf{r}}}(t) \,. \end{aligned}$$

#### Input weights

In order to feed the input data ${\textbf{u}}(t)$ into the reservoir, an input weights matrix ${\textbf{W}}_{in}$ is defined, which determines how strongly each coordinate influences every single node of the reservoir network. In a traditional RC, the elements of ${\textbf{W}}_{in}$ are chosen to be uniformly distributed random numbers within the interval $[-1, 1]$.

In our novel framework we do not choose the elements randomly, but follow a structured approach. Firstly, in order to remove the randomness, for a block-size of *b* we take *b* equally spaced values between [1, 0].

6 $$\begin{aligned} {\textbf{w}} = \left( w_1, w_2, \dots , w_b \right) ^T = \left( 1, \frac{b -2}{b -1}, \dots , \frac{1}{b - 1}, 0\right) ^T \end{aligned}$$

To avoid non-invertible matrices for ridge regression, we take the square root values of all weights ${\textbf{w}}=\left( \sqrt{w_1}, \dots , \sqrt{w_b} \right) ^T$. Then we specifically structure the input matrix so that the different combinations of input data coordinates, also called *features*, are fed separately into the reservoir. In the case of a 3-dimensional system with coordinates ${\textbf{u}}(t)=(x, y, z)^T(t)$ and a nonlinearity order $\eta {=}2$, the input matrix (multiplication) looks like:

7 $$\begin{aligned} {\textbf{W}}_{in} \cdot {\textbf{u}}(t) = \begin{pmatrix} {\textbf{w}} &{} {\textbf{0}} &{} {\textbf{0}}\\ {\textbf{0}} &{} {\textbf{w}} &{} {\textbf{0}}\\ {\textbf{0}} &{} {\textbf{0}} &{} {\textbf{w}}\\ {\textbf{w}} &{} {\textbf{w}} &{} {\textbf{0}}\\ {\textbf{w}} &{} {\textbf{0}} &{} {\textbf{w}}\\ {\textbf{0}} &{} {\textbf{w}} &{} {\textbf{w}}\\ \end{pmatrix} \cdot {\textbf{u}}(t) = \begin{pmatrix} x\\ y\\ z\\ x + y\\ x + z\\ y + z\\ \end{pmatrix}(t) \otimes {\textbf{w}} \end{aligned}$$

where $\otimes$ denotes the tensor product and hence each block represents one feature *f*. For *n*-dimensional data the feature space contains $n_f=2^n{-}2$ elements. Thus, the dimension of the reservoir is $d=n_f \cdot b$.

#### Reservoir

The core of an RC, the reservoir ${\textbf {A}}$, is usually constructed as a sparse Erdős-Rényi random network^27^ with number of nodes *d*. As for the choice of input weights, we choose the reservoir in such a way that each feature remains separate. Therefore, we use a block diagonal matrix J consisting of ones ${\textbf{J}}$ with block size *b*. Thus, each block ${\textbf{J}}_i$ can be directly mapped to a particular feature:

8 $$\begin{aligned} {\textbf{J}} = \begin{pmatrix} {\textbf{J}}_{1} &{} {\textbf{0}} &{} \cdots &{} {\textbf{0}}\\ {\textbf{0}} &{} {\textbf{J}}_{2} &{} \cdots &{} {\textbf{0}}\\ \vdots &{} \vdots &{} \ddots &{} \vdots \\ {\textbf{0}} &{} {\textbf{0}} &{}{\textbf{0}} &{} {\textbf{J}}_{n_f}\\ \end{pmatrix} \longmapsto \begin{pmatrix} {\textbf{J}}_{x} &{} {\textbf{0}} &{} \cdots &{} {\textbf{0}}\\ {\textbf{0}} &{} {\textbf{J}}_{y} &{} \cdots &{} {\textbf{0}}\\ \vdots &{} \vdots &{} \ddots &{} \vdots \\ {\textbf{0}} &{} {\textbf{0}} &{}{\textbf{0}} &{} {\textbf{J}}_{y + z}\\ \end{pmatrix} \, . \end{aligned}$$

Similar to a traditional RC, we scale the spectral radius $\rho ({\textbf{J}})$ of the reservoir to a target spectral radius $\rho ^*$. While the computation of the spectral radius is usually a computationally expensive task^28^ that scales with ${\mathcal {O}}(d^3)$, the computation is no longer necessary for block diagonal matrices of ones ${\textbf{J}}$. This is because the eigenvalues of the matrix are equal to the block size *b*. Thus, the rescaled reservoir is given by:

9 $$\begin{aligned} {\textbf{A}} \equiv \frac{\rho ^*}{\rho ({\textbf{J}})} {\textbf{J}} \longrightarrow \frac{\rho ^*}{b} {\textbf{J}} \,. \end{aligned}$$

Our default target spectral radius is $\rho ^*{=}0.1$.

#### Reservoir states

As in traditional RC, we use a recurrent update equation to capture the dynamics of the system in the so-called reservoir states ${\textbf{r}}(t)$. This would normally require a bounded nonlinear activation function $g(\cdot )$ that captures the nonlinear properties of the data. The activation function (usually the hyperbolic tangent) is applied on an element-by-element basis.

However, as mentioned earlier, we shift the nonlinearity entirely to the readout. Therefore, the time evolution of the states is iteratively determined via:

10 $$\begin{aligned} {\textbf{r}}(t+1)&= g\big ({\textbf{A}} \cdot {\textbf{r}}(t) + {\textbf{W}}_{in} \cdot {\textbf{u}}(t)\big ) \end{aligned}$$

11 $$\begin{aligned}&\longrightarrow {\textbf{A}} \cdot {\textbf{r}}(t) + {\textbf{W}}_{in} \cdot {\textbf{u}}(t) \, . \end{aligned}$$

Due to our choice of architecture, the reservoir states for each feature can be obtained separately:

12 $$\begin{aligned} {\textbf{r}}(t) = \begin{pmatrix} {\textbf{r}}_x\\ {\textbf{r}}_y\\ {\textbf{r}}_z\\ {\textbf{r}}_{x + y}\\ {\textbf{r}}_{x + z}\\ {\textbf{r}}_{y + z}\\ \end{pmatrix}(t) = \begin{pmatrix} {\textbf{r}}_{x, 1}\\ {\textbf{r}}_{x, 2}\\ \vdots \\ {\textbf{r}}_{x, b}\\ {\textbf{r}}_{y, 1}\\ \vdots \\ {\textbf{r}}_{y + z, b} \end{pmatrix}(t)\, . \end{aligned}$$

Hence, we can take all reservoir states belonging to one feature ${\textbf{r}}_f(t)$ — we call them *feature states* — and analyze them separately. This helps us to understand that the reservoir acts as an averaging operator on the feature states:

13 $$\begin{aligned} {\textbf{A}}_f \cdot {\textbf{r}}_f(t) = \left( \frac{\rho ^*}{b} \sum _{i=1}^b {\textbf{r}}_{f_, i}(t) \right) \cdot \mathbf {I}_b = \rho ^* \cdot \bar{{\textbf{r}}}_f(t)\,, \end{aligned}$$

where ${\mathbf {I}}_b$ is a vector of ones of size *b*. Thus, in each iteration step, the feature states are “normalized” to the average of the past feature states $\bar{{\textbf{r}}}_f(t)$ and a varying strength (determined by the input weight) is added to the new feature data *f*(*t*):

14 $$\begin{aligned} {\textbf{r}}_f(t+1) = \rho ^* \cdot \bar{{\textbf{r}}}_f(t) + {\textbf{w}} \cdot f(t)\,. \end{aligned}$$

where *f* can be replaced by any other feature without loss of validity. The average, or “memory”, of each feature is tracked in the last row of the feature states since $w_b{=}0$. Furthermore, this implies that target spectral radius $\rho ^*$ determines how strongly the memory of the data is kept in each iteration step.

#### Readout

While a quadratic readout, i.e., the squared reservoir states ${\textbf{r}}^2$, is often added to a traditional RC to break the symmetry of the activation function^25^, we need the readout to capture the nonlinearity of the data. Therefore, we add even higher orders of nonlinearity to the so-called generalized states $\tilde{{\textbf{r}}}$. For a given degree of nonlinearity $\eta$ they look like the following:

15 $$\begin{aligned} \tilde{{\textbf{r}}} = \left\{ {\textbf{r}}, {\textbf{r}}^2, \dots , {\textbf{r}}^{\eta - 1}, {\textbf{r}}^\eta \right\} \,. \end{aligned}$$

Hence, for a degree of nonlinearity $\eta$ and a block-size of *b*, the number of elements in the readout, which is also the number of variables to be optimized, is:

16 $$\begin{aligned} n_{out} = \left( 2^n - 2\right) \cdot \eta \cdot b = \left( \sum _{k=1}^n \left( {\begin{array}{c}n\\ k\end{array}}\right) - 1 \right) \cdot \eta \cdot b \,, \end{aligned}$$

which we rewrite to binomial coefficients for better comparison. For high-dimensional data with high nonlinearity, the number of variables to be optimized is much smaller than for comparable predictive models such as NG-RC^14^ or SINDy^15^. This is because NG-RC and SINDy require combinations with recurrences. Therefore, the size of their feature space for a nonlinearity degree is $\eta$ (at least):

17 $$\begin{aligned} n_{f} = \sum _{k=1}^\eta \left( {\begin{array}{c}n + k + 1\\ k\end{array}}\right) = \left( {\begin{array}{c}n + \eta \\ n\end{array}}\right) - 1 \, , \end{aligned}$$

which grows much faster for larger *n* and $\eta$ than the expression for $n_{out}$ in Eq. 16.

### Prediction performance measures

When forecasting nonlinear dynamical systems, the goal is not only to exactly replicate the short-time trajectory, but also to reproduce the long-term statistical properties, or climate, of the system.

#### Correlation dimension

To assess the structural complexity of an attractor, we calculate its correlation dimension $\nu$, which measures the fractal dimensionality of the space populated by its trajectory^29,30^. The correlation dimension is implicitly defined by the power-law relationship based on the correlation integral:

18 $$\begin{aligned} C(r)&= \int _{0}^{r} d^n r^{\prime } c({\textbf {r}}^{\prime }) \longrightarrow C(r) \propto r^{\nu } \end{aligned}$$

where *n* is the dimension of the data and $c({\textbf {r}}^{\prime })$ is the standard correlation function. The integral represents the mean probability that two states in the phase space are close to each other at different time steps. This is the case if the distance between the two states is smaller than the threshold distance *r*. For self-similar, strange attractors, this power-law relationship holds for a certain range of *r*, which can be calibrated using the *Grassberger-Procaccia* algorithm^31^. The benefits of this measure are that it is purely data-based, it only needs a small number of data points, and it does not require any knowledge of the underlying governing equations of the system.

#### Lyapunov exponents

Besides its fractal dimensionality, the statistical climate of an attractor is also characterized by its temporal complexity represented by the *Lyapunov* exponents^32^. They describe the average rate of divergence of nearby points in the phase space, and thus measure sensitivity with respect to initial conditions^33^. There is one exponent for each dimension in the phase space. If the system has at least one positive Lyapunov exponent, it is classified as chaotic. Thus, it is sufficient for the purposes in this work to calculate only the largest Lyapunov exponent $\lambda _{max}$:

19 $$\begin{aligned} d(t) = C \cdot e^{\lambda _{max} \cdot t} \, \end{aligned}$$

where *d*(*t*) denotes the distance of two initially nearby states in phase space and the constant *C* is the normalization constant at the initial separation. Thus, instead of determining the full Lyapunov spectrum, we only need to find the largest one as it describes the overall system behavior to a large extent. Therefore we use the *Rosenstein* algorithm^34^.

#### Forecast horizon

To quantify the quality and duration of the short-term prediction of the trajectory we use the forecast horizon $\tau$^12^. It tracks the number of time steps for which the absolute error between each coordinate of the predicted ${\textbf{y}}_{pred}(t)$ and test ${\textbf{y}}_{test}(t)$ data does not exceed the standard deviation of the test data $\varvec{\sigma }\left( {\textbf{y}}_{test}(t)\right)$:

20 $$\begin{aligned} \left| {\textbf{y}}_{pred}(t) - {\textbf{y}}_{test}(t)\right| < \varvec{\sigma }\left( {\textbf{y}}_{test}(t)\right) \,. \end{aligned}$$

We express the forecast horizon in units of the Lyapunov time by multiplying it with the discretization and maximum Lyapunov exponent of the test data $\tau \cdot dt \cdot \lambda _{max}^{test}$. This measure is intended to evaluate how long a prediction can reproduce the actual trajectory before the chaotic nature of the system leads to an exponential divergence.

### Dynamical systems

We apply our model to a number of synthetic chaotic systems. In our analyses, we focus on the following three due to their specific properties in terms of nonlinearity.

#### Lorenz

As it is common in RC research^35,36^ we use the Lorenz system which was initially proposed for modeling atmospheric convection^37^:

21 $$\begin{aligned} \begin{aligned} \frac{d x}{d t}&= \sigma \cdot (y-x)\\ \frac{d y}{d t}&= x \cdot (\rho - z) - y \\ \frac{d z}{d t}&= x \cdot y - \beta \cdot z \, , \end{aligned} \end{aligned}$$

where the standard parametrization for chaotic behavior is $\sigma {=}10$, $\rho {=}28$, and $\beta {=}8/3$.

#### Halvorsen

As in Hertreux and Räth^25^ we use the Halvorsen system^38^ for our analyses, which has a cyclic symmetry and, unlike to the Lorenz system, only has nonlinearities without interaction of coordinates:

22 $$\begin{aligned} \begin{aligned} \frac{d x}{d t}&= a \cdot x - b \cdot y - b \cdot z - y^2 \\ \frac{d y}{d t}&= a \cdot y - b \cdot z - b \cdot x - z^2 \\ \frac{d z}{d t}&= a \cdot z - b \cdot x - b \cdot y - x^2 \, , \end{aligned} \end{aligned}$$

where $a{=}1.3$ and $b{=}4$ are the standard parameters.

#### Rabinovich–Fabrikant

In order to test whether our model works also for systems entailing cubic nonlinearities, we analyze the Rabinovich-Fabrikant system^39^:

23 $$\begin{aligned} \begin{aligned} \frac{d x}{d t}&= y \cdot (z - 1 + x^2) + \gamma \cdot x \\ \frac{d y}{d t}&= x \cdot (3 \cdot z + 1 - x^2) + \gamma \cdot y \\ \frac{d z}{d t}&= -2 \cdot z \cdot (\alpha + x \cdot y)\, , \end{aligned} \end{aligned}$$

where $\alpha {=}0.14$ and $\gamma {=}0.1$ are the standard parameters.

#### Simulating and splitting data

Since we compare our model with NG-RC and SINDy, we use the same data setup as the original works^14,15^. Hence, we solve the differential equations of the systems using the *Runge-Kutta* method^40^ with a discretization of $dt{=}0.025$ in order to ensure a sufficient manifestation of the attractor.

We discard the initial transient of $T_{transient}{=}50000$, use the next $T_{train}{=}400$ steps for training, then skip $T_{skip}{=}10000$ steps, and use the remaining $T_{test}{=}10000$ for testing the prediction. Hence in total we simulate $T{=}70400$ steps.

To get robust results, we also vary the starting points on the attractor by using the rounded last point of one data sample as the starting point for the next. The initial starting points for the Lorenz, Halvorsen, and Rabinovich-Fabrikant systems are $(-14, -20, 25)$, $(-6.4, 0, 0)$, and $(-0.4, 0.1, 0.7)$, respectively.

### Comparable prediction models

We compare our novel RC to other models designed for predicting dynamical systems. We briefly describe them in the following.

#### Traditional reservoir computer

For the traditional RC architecture we choose an Erdős-Rényi network of dimension $d{=}600$ with a target spectral radius $\rho ^*$ and a quadratic readout. This equals 1200 variables per coordinate to be optimized. In order to get robust results we repeat the prediction for $1000$ realizations and take the average of the prediction measures.

#### Next generation reservoir computer

The next generation reservoir computer (NG-RC) developed by Gauthier et al.^14^ is a so-called nonlinear vector autoregression (NVAR) machine and thus, does not require a reservoir. It solely needs the feature vector, which consists of time-delay observations of the data and nonlinear functions of these observations. The resulting output weights can be used to construct the governing equations of the data. We use the standard setting with time delays $k{=}2$ and skips $s{=}1$.

#### Sparse identification of nonlinear dynamics

Sparse identification of nonlinear dynamics (SINDy)^15^ discovers the underlying dynamical system of data by learning its governing equations through sparse regression. It is similar to NG-RC, but uses an iterative approach to filter only relevant features. We use the standard parametrization and the official Python package PySINDy^41,42^.

**Table 1:** Minimal setup for different chaotic systems. We vary the parameters of the training data and the RC architecture to find the minimal setup for different chaotic systems. To do this, we compute the relative errors of the Lyapunov exponent and the correlation dimension for 100 different attractor starting points. The minimum setup is defined as the setup where the average relative errors of the Lyapunov exponent and the correlation dimension are both ${<}10^{-2}$. This ensures that the long-term climate of the chaotic system is reliably reproduced. In this table, we denote the parameters of the data setup (columns $1{-}2$) and RC architecture (columns $3{-}5$). The last 3 columns denote the mean and standard deviations of the forecast horizon for the different prediction models. The governing equations can be found in the respective references.

	Training Data		Novel Architecture			Forecast Horizon $\tau$			
System	$T_{train}$	*dt*	*b*	$\rho ^*$	$\eta$	Novel	Trad.	NG-RC	SINDy
Halvorsen	300	0.01	3	0.1	2	$498{\pm }34$	$231{\pm }47$	$249{\pm }27$	$335{\pm }37$
Rabi.-Fabr.	300	0.01	3	0.1	3	$261{\pm }23$	$168{\pm }36$	$89{\pm }12$	$107{\pm }11$
Aizawa (^43^)	300	0.01	3	0.1	4	$193{\pm }16$	$131{\pm }27$	$76{\pm }9$	${65\pm }7$
Dadras-Momeni (^44^)	300	0.01	3	0.1	2	$423{\pm }25$	$228{\pm }41$	$259{\pm }19$	${248\pm }21$
Rössler (^45^)	300	0.01	3	0.1	2	$781{\pm }51$	$301{\pm }72$	$332{\pm }40$	${401\pm }55$
Four wing (^46^)	300	0.01	3	0.1	2	$1497{\pm }39$	$1135{\pm }68$	$659{\pm }28$	$712{\pm }31$
Chen (^47^)	300	0.01	3	0.1	2	$922{\pm }41$	$880{\pm }72$	$750{\pm }36$	$812{\pm }41$

## Data Availability

The datasets used and/or analyzed during the current study available from the corresponding author on reasonable request.
